# Supplementary material for: Next-generation plasmids for transgenesis in zebrafish and beyond
Source: Development. 2023 Apr 19;150(8):dev201531. doi: 10.1242/dev.201531 (PMC10263156; doi:10.1242/dev.201531)
Supplement: Supplementary information [file develop-150-201531-s1.pdf]

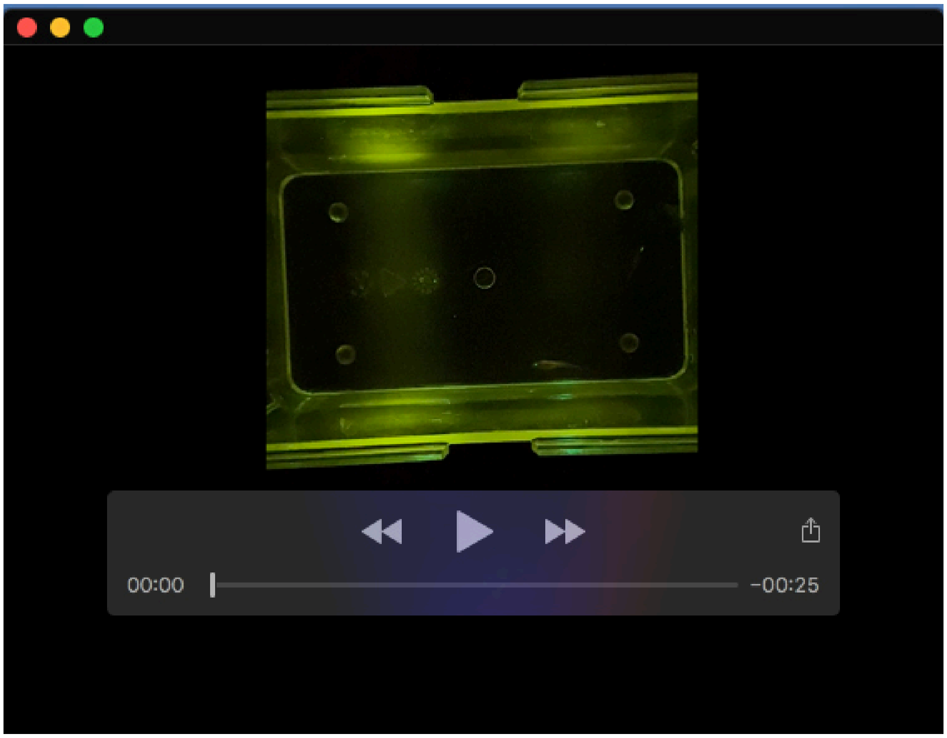

Movie 1. Adult *exorh:EGFP*-transgenic zebrafish observed using a NightSEA hand-held UV flashlight and UV filter glasses ("goggles"), demonstrating the persistent pineal gland expression of the reporter and the simple detection.
