# Supplementary material for: Next-generation plasmids for transgenesis in zebrafish and beyond
Source: Development. 2023 Apr 19;150(8):dev201531. doi: 10.1242/dev.201531 (PMC10263156; doi:10.1242/dev.201531)
Supplement: Supplementary file 1 [file develop-150-201531_review_history.pdf]

Next-generation plasmids for transgenesis in zebrafish and beyond

Cassie L. Kemmler, Hannah R. Moran, Brooke F. Murray, Aaron Scoresby, John R. Klem, Rachel L. Eckert, Elizabeth Lepovsky, Sylvain Bertho, Susan Nieuwenhuize, Sibylle Burger, Gianluca DAgati, Charles Betz, Ann-Christin Puller, Anastasia Felker, Karolina Ditrychova, Seraina Bötschi, Markus Affolter, Nicolas Rohner, C. Ben Lovely, Kristen M. Kwan, Alexa Burger and Christian Mosimann
DOI: 10.1242/dev.201531

Editor: Steve Wilson

Review timeline

Original submission:	14 December 2022
Editorial decision:	30 January 2023
First revision received:	20 February 2023
Accepted:	10 March 2023

Original submission

First decision letter

MS ID#: DEVELOP/2022/201531

MS TITLE: Next-generation plasmids for transgenesis in zebrafish and beyond

AUTHORS: Cassie L. Kemmler, Hannah R. Moran, Brooke F. Murray, Aaron Scoresby, John R. Klem, Rachel L. Eckert, Elizabeth Lepovsky, Sylvain Bertho, Susan Nieuwenhuize, Sibylle Burger, Gianluca DAgati, Charles Betz, Ann-Christin Puller, Anastasia Felker, Karolina Ditrychova, Seraina Botschi, Markus Affolter, Nicolas Rohner, C. Ben Lovely, Kristen M. Kwan, Alexa Burger, and Christian Mosimann

I have now received all the referees' reports on the above manuscript, and have reached a decision. The referees' comments are appended below, or you can access them online: please go to BenchPress and click on the 'Manuscripts with Decisions' queue in the Author Area.

As you will see, thereferees likeyour manuscript but have some suggestions for improvement. I will be happy receive a revised version of the manuscript after you have revised the manuscript along the lines suggested.

Please attend to all of the reviewers' comments and ensure that you clearly highlight all changes made in the revised manuscript. Please avoid using 'Tracked changes' in Word files as these are lost in PDF conversion. I should be grateful if you would also provide a point-by-point response detailing how you have dealt with the points raised by the reviewers in the 'Response to Reviewers' box. If you do not agree with any of their criticisms or suggestions please explain clearly why this is so.

Reviewer 1

Advance summary and potential significance to field

Kemmler et al. report the production of a new collection of Gateway compatible vectors for transgenesis in zebrafish and some other species, such as the Mexican cavefish. This work represents an expansion of the original Tol2Kit (Kwan et al., Developmental Dynamics, 2007) to

© 2023. Published by The Company of Biologists under the terms of the Creative Commons Attribution License (https://creativecommons.org/licenses/by/4.0/).

expand existing functionality, including additional options for fluorophore and transgenesis marker selection, and to introduce new functionality including vectors for enhancer testing and for p2a-based protein co-expression.

Comments for the author

As a resource, this collection will undoubtedly be of great value to the zebrafish research community. The original Tol2Kit is a popular and widely used resource for quick and easy transgene production, and the new and expanded functions of this kit will be of use to many research groups.

As a scientific advance, this work does not appear to break any new ground. Rather, the authors have taken a series of elements & functions already in use in the fish (e.g. the mouse beta-globin minimal promoter for enhancer testing, the p2a element for protein co-expression, the pineal expression-promoting element for pineal organ expression) and compiled them into an organized, accessible, easy-to-use toolkit. The efficacy of each element is based on prior literature. This lack of scientific novelty does not detract from the collection's value as a resource for the fish community, which is considered major. The authors are in the process of making the plasmids available via Addgene. Thus, the manuscript satisfies the criteria for a Resource paper in Development.

For the most part, the results reported in this manuscript are convincing, and the authors have satisfactorily shown that their vectors function as intended, although in most cases this was already known. A few areas in which results/conclusions require additional support or should be revised are:

Major Points

1. Regarding use of the mHbb-bt minimal promoter: the authors mention the risk of such a promoter being sensitive to enhancers in the vicinity of the transgene insertion, and reference other publications to suggest that this promoter poses little such risk, they do not test the extent to which their miniprom constructs are susceptible to such positional effects. This risk is of particular concern when testing putative enhancers of unknown function, which the authors did not do. The authors should 1) share additional data showing the probability of such effects by, for instance, cloning a nonfunctional sequence upstream of the miniprom:eGFP and assessing the frequency of expression in transient transgenic injections or 2) amend the text to acknowledge the uncertainty of such a risk with these constructs.

2. Regarding co-expression using the p2a element (Fig. 4B-E): the authors show that the genes on either side of the p2a appear to be reliably coexpressed; however, in figure 4B there are several mCherry punctae that do not have associated EGFP signal. It is unclear to me whether this represents a failure of co-expression in some cells (which would be concerning) or, as I suspect, that these mCherry punctae are not in fact nuclei but just specs of fluorescence. The authors should make note of these punctae and provide an explanation for them.

3. Regarding stoichiometric expression using the p2a element (Fig. 4E): the authors provide qualitative analysis of a single image to suggest the expression of genes on either side of the p2a element is stoichiometric (an inherently quantitative term). While this image does suggest some proportionality of expression, quantifying and comparing membrane and nuclear GFP fluorescence intensity in cells with varying levels of expression would greatly strengthen the authors' claims.

Reviewer 2

Advance summary and potential significance to field

In this manuscript, Kemmler and colleagues present a toolbox addition to the existing Tol2 transgenesis platform widely used by the zebrafish community. These include a few more fluorophores (mCerulean and mApple), options to use these fluorophores localized to nucleus or membrane or as part of a bicistronic reporter using the viral 2A peptide. The whole system is based on the same Gateway technology as the existing Tol2 kit and provides a useful extension to it.

© 2023. Published by The Company of Biologists under the terms of the Creative Commons Attribution License (https://creativecommons.org/licenses/by/4.0/).

In addition, they test and validate an exohr promoter-driven cassette as a new transgenesis marker to add to the existing cryaa or myl7 reporters. This is validated in both zebrafish and Astianax and provide a welcome alternative to cryaa- or myl7-based transgenic markers. Overall, this is a very useful addition to existing tools for transgenesis and I expect they will be a very welcome resource and very much used by the community (particularly once readily available from Addgene). I salute the authors for submitting all sequences and maps.

There are some issues that I would encourage the authors to address, see below:

1.It would be good to have a summary table of all the constructs as a figure (or a first panel in Fig1), separating the constructs by type – it would make it easier to grasp all the different constructs available.

2.In the first description of mCerulean as an alternative marker, there is no consideration regarding detection of mCerulean using epifluorescence – can it be distinguished from GFP then or will common fluorescence microscopes show some 'bleedthrough' fluorescence in the GFP detection channel? It would be useful to add a note of caution in that regard.

3.Related to that, how is the overlap between detection of GFP and mApple? Can they be used as a double transgenic in epifluorescence without detecting the GFP in the mApple channel? Or best to image these fluorophore combinations only in confocal microscopy?

4.The desma-mApple-exohr-GFP and desma-mCerulean-cryya-Venus vectors are going to be very useful to use as vectors for testing enhancer elements for labs that use 'traditional' cloning instead of Gateway. However, mCerulean is generally more difficult to detect than GFP (less bright). It would be very helpful/useful if the authors were to expand this set slightly to include a green version of these plasmids (e.g. desma-emerald green-exohr-GFP)

Comments for the author

1.In the description of common minimal promoters used for transgenesis, there is no mention of the E1b minimal promoter (from e.g. Birnbaum et al, 2012, Genome Res or Chong-Morrison et al, biorxiv)
2.Fig 1 – needs labelling of the various structures in the panels
3.Fig 2 – Fig legend text for panels B and C are mentioned in the reverse order.
Also, it would be good to label the unspecific fluorescence in panel A
4.Discussion – the pAP02 p3E MCS 3' entry vector enabling downstream cloning of enhancers is not really mentioned in the results section, or shown whether it can work/drive fluorescence of a reporter – did you test this design?
5.Methods – concentrations of plasmid and RNA are expressed as ng/ml (mililiter) – did you mean ng/µl (microliter)?

<u>Reviewer 3</u>

Advance summary and potential significance to field

This paper reports a series of new transgenesis vectors suitable for use in fish.
Their deployment in the miniTol2 vector makes these accessible to most current labs. The selected images are well-presented. The text language strongly indicates these lines show Mendelian expression patterns, an issue they argue has prevented prior tools from more regular use in the field. These vectors will likely be well-used by the research community and represent a valuable asset.
This data represents a substantial body of work. All comments below could be addressed by textual changes, but some might be better updated with data should the authors have collected during the course of the work generating these lines.

Comments for the author

Some major points:

© 2023. Published by The Company of Biologists under the terms of the Creative Commons Attribution License (https://creativecommons.org/licenses/by/4.0/).

1. Data supporting claims around the Mendelian nature of the expression of these lines is just not presented. That will require assessing more than a single example for each, as the use of random integration means each construct example they include could come from selection bias. Alternatively, the authors could reduce their claims to the line examples they present. But please be specific on how many lines you studied for each listed construct.

2. Are the overall transgenesis rates the same for all vectors?

3. They deploy an array of different fluorophores and argue these do not bleed into more traditional colors such as GFP. These claims are largely unsubstantiated - there are relatively few double transgenics for example. As noted in issue (1), this could be addressed by either adding data or by reducing claims as written in the text.

4. The pineal gland transgenic reporter is interesting, but it actually looks difficult to photograph. How is it in use on stereo dissecting fluorescent screening scopes?

5. Providing clear data on the limitation of IRES use would be good for the field. Are there good tangible examples the authors can provide as they developed the prior transgenesis kit that heavily depended on IRES expression?
Some individual reports are included. But a comprehensive collection on this topic would be an excellent use of space within this manuscript.

Minor:
Note: adding 2A peptide sequence can reduce the expression of the upstream ORF.

Textual
I found the number of missing citations substantial to make reading the paper difficult to fully assess. Some examples are below. Might be simpler to find a few reviews and focus more on the science in the paper. I point out a few critical ones, but none are more important than those from Fisher 2006.

Intro, first paragraph
The selection of examples showcasing transgenic constructs seems rather arbitrarily selected and omits a variety of classics (such as the first GATA GFP from Shuo Lin).

Paragraph two - Tol2 was first harnessed by Dr Koga’s lab, isolated from medaka.
That citation is missing here but found later in the paper (Koga 1996).

Last paragraph - the selection of citations seems off as well. Amsterdam 1995 is a great paper, but it’s not generating a lot of transgenics with expression. And if the focus is on first transgenics, many other citations are missing. Might be simpler to cite far fewer rather than have a broader aperture and clearly miss critical papers.

Results
First paragraph: also many citations missing, including two Fisher et al 2006 key papers. And papers prior to Fisher conducting targeted enhancer trapping in zebrafish etc.

First revision

Author response to reviewers' comments

Reviewer 1 Advance Summary and Potential Significance to Field:

Kemmler et al. report the production of a new collection of Gateway compatible vectors for transgenesis in zebrafish and some other species, such as the Mexican cavefish. This work represents an expansion of the original Tol2Kit (Kwan et al., Developmental Dynamics, 2007) to expand existing functionality, including additional options for fluorophore and transgenesis marker

© 2023. Published by The Company of Biologists under the terms of the Creative Commons Attribution License (https://creativecommons.org/licenses/by/4.0/).

selection, and to introduce new functionality, including vectors for enhancer testing and for p2a-based protein co-expression.

Reviewer 1 Comments for the Author:
As a resource, this collection will undoubtedly be of great value to the zebrafish research community. The original Tol2Kit is a popular and widely used resource for quick and easy transgene production, and the new and expanded functions of this kit will be of use to many research groups. As a scientific advance, this work does not appear to break any new ground. Rather, the authors have taken a series of elements & functions already in use in the fish (e.g. the mouse beta-globin minimal promoter for enhancer testing, the p2a element for protein co-expression, the pineal expression-promoting element for pineal organ expression) and compiled them into an organized, accessible, easy-to-use toolkit. The efficacy of each element is based on prior literature. This lack of scientific novelty does not detract from the collection's value as a resource for the fish community, which is considered major. The authors are in the process of making the plasmids available via Addgene. Thus, the manuscript satisfies the criteria for a Resource paper in Development.

We thank the reviewer for their supportive assessment of our efforts. Indeed, the collection of plasmids was driven by pre-existing and at times well-validated components. We hope our combined vector collection and application examples will motivate more labs in the field to expand the number of transgenic lines available in our model.

For the most part, the results reported in this manuscript are convincing, and the authors have satisfactorily shown that their vectors function as intended, although in most cases this was already known. A few areas in which results/conclusions require additional support or should be revised are:

Major Points

1. Regarding use of the mHbb-bt minimal promoter: the authors mention the risk of such a promoter being sensitive to enhancers in the vicinity of the transgene insertion, and reference other publications to suggest that this promoter poses little such risk, they do not test the extent to which their miniprom constructs are susceptible to such positional effects. This risk is of particular concern when testing putative enhancers of unknown function, which the authors did not do. The authors should 1) share additional data showing the probability of such effects by, for instance, cloning a nonfunctional sequence upstream of the miniprom:eGFP and assessing the frequency of expression in transient transgenic injections or 2) amend the text to acknowledge the uncertainty of such a risk with these constructs.

The reviewer raises a central issue with numerous enhancer testing setups applied in zebrafish and other models. The mHbb-bt minimal promoter had been previously used and validated by Woolfe et al., 2004, and Tamplin et al., 2011 for uncovering enhancers active in distinct tissues. A key benefit of the *mHbbt-bt* minimal promoter is its exceedingly small size. Of note, the mouse and chick beta-globin minimal promoters are widely used in the mouse community to generate transgenic reporters since decades.

In our own work, we have uncovered several new regulatory sequences by F0 evaluation and have generated dozens of transgenics using the minimal promoter following our routine procedures of isolating multiple independent founders (Felker & Mosimann, 2016). To avoid bias, we always check several clutches from at least three independent F1 founders and we have amended the text in the methods. Our prior use of the minimal promoter are documented in our previous publications (for instance Mosimann et al., 2015; Kaufman et al., 2016; D'Agati et al., 2018; Kirchgeorg et al., 2018; Prummel et al., 2019; Prummel et al., 2022; and ongoing projects). We hoped to avoid exuberant self-citation of our work in this regard in the manuscript and aimed to document the promoter's use concisely for the readers of our manuscript.

As additional example, together with our *HOPX*^{4q12N} regulatory element located on chr. *4q12* (as seen in Fig. 2A, Fig. 6C), we have cloned 13 more putative regulatory sequences in the vicinity of the human *HOPX* gene that we expected to show reporter expression in the notochord. When we tested these 14 regulatory elements in F0 transient reporter assays using our *minprom:mCerulean*

© 2023. Published by The Company of Biologists under the terms of the Creative Commons Attribution License (https://creativecommons.org/licenses/by/4.0/).

reporter (co-injected with *ubi:mCherry* plasmid as injection control) 4/14 putative regulatory elements were inactive and did not show any notochord activity (regulatory element J: n=0/98, K: n=0/68, M: n=0/97, O: n=0/68) nor activity anywhere else in the zebrafish embryo. While anecdotal (manuscript depicting this data in preparation), we hope this additional info helps to address the reviewer's concern.

We nonetheless cannot exclude the influence of position effects from random integrations on this promoter – which is however true for any other minimal promoter as well. To document how versatile and inert the *mHbb- bt* minimal promoter is, we now included F0-based testing of its baseline activity using *T7-minprom- EGFP,cryaa:Venus* (Fig. 1). These experiments serve as background control and we hope that users will include such zero-expression comparisons from their own enhancer testing in future publications.

2. Regarding co-expression using the p2a element (Fig. 4B-E): the authors show that the genes on either side of the p2a appear to be reliably coexpressed; however, in figure 4B there are several mCherry punctae that do not have associated EGFP signal. It is unclear to me whether this represents a failure of co-expression in some cells (which would be concerning) or, as I suspect, that these mCherry punctae are not in fact nuclei, but just specs of fluorescence. The authors should make note of these punctae and provide an explanation for them.

We appreciate reviewer's keen eye in noting of the punctae in our transient injection experiments.

The punctae may arise due to different factors. First, these were transient injections, and there is likely sporadic cell death, which may be positive in the mCherry channel. This is likely to account for the smaller punctae that are apparent in the image.

Second, some punctae are larger or represent a partial nucleus via optical sectioning. In these cases, there were likely to be differences in how each channel was adjusted for brightness and contrast; in 4B in particular, because the membrane fluorescence (EGFP) is distributed throughout the entire plasma membrane, it may appear dimmer than the concentrated nuclear mCherry fluorescence.

Closer examination of the image reveals that if the EGFP signal is increased for brightness, there is signal where there are larger punctae that are likely to be nuclei.

To address this concern, we now have replaced the images in Figure 4B-D.

3. Regarding stoichiometric expression using the p2a element (Fig. 4E): the authors provide qualitative analysis of a single image to suggest the expression of genes on either side of the p2a element is stoichiometric (an inherently quantitative term). While this image does suggest some proportionality of expression, quantifying and comparing membrane and nuclear GFP fluorescence intensity in cells with varying levels of expression would greatly strengthen the authors' claims.

Stoichiometry infers a quantitative measure, and Kim et. al. (https://www.ncbi.nlm.nih.gov/pmc/articles/PMC3084703/) showed convincingly that 2A peptides provide stoichiometric expression (e.g. equal protein levels of the 5' and 3' encoded ORFs), in particular P2A. In addition, 2A peptides have been extensively adapted for the *in vitro* reprogramming of iPS and ES cells to various cell fates such as cardiomyocytes, where equal levels of individual transcription factors is key (e.g. https://pubmed.ncbi.nlm.nih.gov/25416133/; https://doi.org/10.1161/CIRCRESAHA.114.305696).

Nonetheless, as suggested by the reviewer, we quantified fluorescence intensity of the membrane and nuclear EGFP, comparing the 2A and IRES, and added the quantifications as graph with the results as panel 2G.

The quantification was done by drawing a line across the cell in ImageJ/FIJI and measuring the fluorescence intensity profile along the line. Signal maxima in the nucleus and at the membrane were recorded; measurements were not used if signal was saturated. The fluorescence intensity ratio (nucleus:membrane) was calculated for each individual cell. This is not an ideal measurement, as the EGFPCAAX signal is distributed around the entire cell surface, while the

© 2023. Published by The Company of Biologists under the terms of the Creative Commons Attribution License (https://creativecommons.org/licenses/by/4.0/).

nlsEGFP signal is concentrated in the nucleus, such that stoichiometric expression would not be expected to yield equal fluorescence intensities at these different subcellular locations. Despite this, we proceeded, as this would at least provide a comparison of fluorescence intensity ratio between the 2A and the IRES.

As now shown in Fig. 4E, when using the 2A peptide, the average nuclear:membrane fluorescence intensity ratio is 1.90±0.45 (see Fig. 4G); a perfectly stoichiometric signal would be 1 (dotted line). In contrast, in Fig. 4F, when using the IRES, the average nuclear:membrane fluorescence intensity ratio is 4.91±1.75 (see Fig. 4G), indicating higher accumulation of nlsEGFP relative to EGFPCAAX in the IRES condition. As this measurement does not strictly evaluate stoichiometry, we have edited out language suggesting that our constructs confer stoichiometric expression.

Reviewer 2 Advance Summary and Potential Significance to Field:
In this manuscript, Kemmler and colleagues present a toolbox addition to the existing Tol2 transgenesis platform widely used by the zebrafish community. These include a few more fluorophores (mCerulean and mApple), options to use these fluorophores localized to nucleus or membrane or as part of a bicistronic reporter using the viral 2A peptide. The whole system is based on the same Gateway technology as the existing Tol2 kit and provides a useful extension to it.

In addition, they test and validate an exohr promoter-driven cassette as a new transgenesis marker to add to the existing cryaa or myl7 reporters. This is validated in both zebrafish and Astianax and provide a welcome alternative to cryaa- or myl7-based transgenic markers. Overall, this is a very useful addition to existing tools for transgenesis and I expect they will be a very welcome resource and very much used by the community (particularly once readily available from Addgene). I salute the authors for submitting all sequences and maps.

We appreciate the reviewer’s encouraging take on our manuscript. We strive to have our tools widely available, and the Addgene deposit is nearly complete. We will also be ready to send the plasmids to labs that cannot afford or access Addgene in the future.

There are some issues that I would encourage the authors to address, see below:
1. It would be good to have a summary table of all the constructs as a figure (or a first panel in Fig 1), separating the constructs by type – it would make it easier to grasp all the different constructs available.

This is a great suggestion. We have moved the table from the Methods into the main text as formatted Table 1.

2. In the first description of mCerulean as an alternative marker, there is no consideration regarding detection of mCerulean using epifluorescence – can it be distinguished from GFP then or will common fluorescence microscopes show some ‘bleedthrough’ fluorescence in the GFP detection channel? It would be useful to add a note of caution in that regard.

We appreciate the reviewer’s concern, which is a detail we have been dealing with and thinking about ever since introducing blue fluorophores into our routine transgene clonings.

Standard dissecting scopes with generally less stringent filters will definitely show mCerulean (or really most blue fluorophores) in the GFP/green channel. While the hue difference is discernible with a little practice, complete separation requires a confocal setup. In practical terms, use of dedicated blue filters on the filter wheel together with green and red filters is highly recommended; however we know of several labs who do their routine blue sorting using GFP filters before moving to confocal imaging for channel separation if multi- color imaging is needed.

We have now added the following text to the results when discussing mCerulean imaging:
“Of note, mCerulean is easily visible on a standard dissecting scope with epifluorescence equipment, ideally using blue fluorescence filters and excitation spectra. mCerulean is also excited and detectable by standard setups used for GFP fluorescence, requiring care to avoid misinterpreting mCerulean versus GFP signal in double-transgenic embryos.”

© 2023. Published by The Company of Biologists under the terms of the Creative Commons Attribution License (https://creativecommons.org/licenses/by/4.0/).

To help users to recapitulate the exact imaging setup that we have been using for the majority of the shown images, we now collected all the technical specs of our dissecting scope setup. We have added more details to our Methods to now state:
"Dissecting scope fluorescence imaging was performed on a Leica M205FA with a DFC450 C camera and 1.0x PlanApo M-Series objective, illuminated with a TL5000 light base and CoolLED pE-300white Illumination System. We use the following Leica filter sets for fluorescence: ET GFP 10447408, ET CFP 10447409, and ET DSR 10447412 for imaging and sorting, and TXR LP 10450590 for routine sorting of red fluorescence. Laser scanning confocal microscopy was performed on a Zeiss LSM880 following embedding in E3 with 1% low- melting-point agarose (Sigma Aldrich, A9045) on glass bottom culture dishes (Greiner Bio-One, Kremsmunster, Austria, 627861). Images were collected with a 10/0.8 air-objective lens with all channels captured sequentially with maximum speed in bidirectional mode, with the range of detection adjusted to avoid overlap between channels. Maximum projections of acquired Z-stacks were made using ImageJ/Fiji69 and cropped and rotated using Adobe Photoshop 2022."

3. Related to that, how is the overlap between detection of GFP and mApple? Can they be used as a double transgenic in epifluorescence without detecting the GFP in the mApple channel? Or best to image these fluorophore combinations only in confocal microscopy?

We do not believe there should be concern for overlap, as mApple is red-fluorescent. We have added further indications for this new fluorophore throughout the text.

4. The desma-mApple-exohr-GFP and desma-mCerulean-cryya-Venus vectors are going to be very useful to use as vectors for testing enhancer elements for labs that use 'traditional' cloning instead of Gateway. However, mCerulean is generally more difficult to detect than GFP (less bright). It would be very helpful/useful if the authors were to expand this set slightly to include a green version of these plasmids (e.g. desma-emerald green-exohr-GFP)

In our experience, mCerulean is easily as bright as EGFP depending on the individual transgenes harboring these fluorophores. In the exorh constructs, mCerulean has been exceedingly bright and easily detectable with both dissecting scopes and confocal microscopy (or light sheet). We have made at least a dozen stable transgenic lines using mCerulean, and neither line is less bright than a typical EGFP line following selection.
This robustness is why we have fully embraced mCerulean as now common fluorophore in our labs.

Nonetheless, the green/green vector suggested by the reviewer is a great idea. To support gene regulation work of labs that do not have the means to invest into blue filter sets or want to simplify the process, we have now generated an EGFP based version of our tester vector as *desmaMCS:EGFP,exorh:mCherry*. We also added other color iterations of this vector to provide an even more versatile set for enhancer discovery, injection testing, and more (Table 1, Fig. 6). We are in the process of depositing these new vectors with AddGene as addendum to the collection.

Reviewer 2 Comments for the Author:

1. In the description of common minimal promoters used for transgenesis, there is no mention of the E1b minimal promoter (from e.g. Birnbaum et al, 2012, Genome Res or Chong-Morrison et al, biorxiv)

We have added the E1b minimal promoter to the list now, and cite what we believe to be its first use in zebrafish (as also cited by Birnbaum et al., 2012) with Li et al., 2010 (https://www.sciencedirect.com/science/article/pii/S0012160609012780).

2. Fig 1 – needs labelling of the various structures in the panels

We have added asterisks and arrowheads to panels C,D to match the text in the Figure legend describing the structures.

3. Fig 2 – Fig legend text for panels B and C are mentioned in the reverse order. Also, it would be good to label the unspecific fluorescence in panel A.

© 2023. Published by The Company of Biologists under the terms of the Creative Commons Attribution License (https://creativecommons.org/licenses/by/4.0/).

We have now revised and edited the figure legends.

The “unspecific” fluorescence is auto-fluorescence of skin or pigment cells. We mention this in the first paragraph of “Components for fluorescent reporter generation”.

We further noticed unspecific expression of the reporter in the yolk when using *pCB24* and *pCB59*. It seems that the *crybb1* promoter in addition to driving mKate2 for example also drives *mCerulean*, which fades in adults and is therefore not a concern in germline-transmitted insertions and established stable lines.

4. Discussion – the pAP02 p3E MCS 3’ entry vector enabling downstream cloning of enhancers is not really mentioned in the results section, or shown whether it can work/drive fluorescence of a reporter – did you test this design?

We appreciate the reviewer’s comment – 3’ entry vectors are the most-forgotten vectors in Gateway tool kits! We have so far not had any application for a 3’ enhancer for testing. In this manuscript, we have used *pAP02 p3E MCS* to clone *pGD003 p3E-ubbpA* as shown in Fig. 2C and have now described this as such to introduce this versatile 3’ vector:
“To expand the available 3’ UTRs for transgene assembly, we isolated a 516 bp fragment of the 3’ UTR of the zebrafish *ubiquitin B* (*ubb, ubi*) gene. We designed *pA02* as *p3E* vector with versatile multiple cloning site to enable the generation of new 3’ vectors by restriction enzyme cloning; transferring the *ubb* 3’ region into *pAP02* resulted in *p3E_ubb-polyA*.”

We also noted now in the discussion that *pAP02* can be used for cloning barcode cassettes (i.e. *GESTALT*) or new polyadenylation sequences. We hope our construct can put the utility of 3’ trailers on everyone’s radar.

5. Methods – concentrations of plasmid and RNA are expressed as ng/ml (mililiter) – did you mean ng/µl (microliter)?

We thank the reviewer for their eye for details - it is indeed microliter (the m was a badly chosen placeholder…) and we have corrected this.

Reviewer 3 Advance Summary and Potential Significance to Field:

This paper reports a series of new transgenesis vectors suitable for use in fish. Their deployment in the miniTol2 vector makes these accessible to most current labs. The selected images are well-presented. The text language strongly indicates these lines show Mendelian expression patterns, an issue they argue has prevented prior tools from more regular use in the field. These vectors will likely be well-used by the research community and represent a valuable asset. This data represents a substantial body of work. All comments below could be addressed by textual changes, but some might be better updated with data should the authors have collected during the course of the work generating these lines.

We appreciate the reviewer’s encouraging comment on our manuscript. We cannot find any mention in our manuscript where we claim that issues with Mendelian transmission of transgenes have prevented the use of prior tools. The issue with Mendelian segregation (or lack thereof) depends on quality control and transgene selection, which we would argue lies in the hands of the users. Our vectors are meant to add and augment the tremendous resources already available in the field.

Reviewer 3 Comments for the Author:

Some major points:
1. Data supporting claims around the Mendelian nature of the expression of these lines is just not presented. That will require assessing more than a single example for each, as the use of random integration means each construct example they include could come from selection bias. Alternatively, the authors could reduce their claims to the line examples they present. But please be specific on how many lines you studied for each listed construct.

© 2023. Published by The Company of Biologists under the terms of the Creative Commons Attribution License (https://creativecommons.org/licenses/by/4.0/).

We do understand the reviewer's point. High-quality, reproducible transgenesis to create lines that are of future use to our and others' research is a key goal of our work. We have now added additional text in the Methods and text to outline and specify this. Notably, the variability the reviewer eludes to is focused on the used regulatory elements; besides the *exorh* and *desma* reporters, for which we have ample quantifications of their faithful expression, the other regulatory elements shown are included as examples, several of which have a) either been used in prior work or b) have been validated in the same rigorous way we generate all our transgenics (see also Felker & Mosimann, 2016, for details). In the end, selection for "good" lines with faithful expression is in the hands of the users and individual labs selecting their lines.

The added text now reads: "Our transgenesis rate is routinely between 15-50% percent for each injected Tol2 transgenesis vector. We investigate several clutches from at least three independent F1 founders. All presented stable transgenic lines are at least F3 generation or beyond and were selected to transmit the transgenes at Mendelian ratios as outlined in Felker and Mosimann, 2016."

We also added to the introduction the following statement: "Whenever showing germline-transmitted, stable transgenic lines, we selected multiple founders and quality-controlled for expected Mendelian ratios in subsequent generations; we have been observing most transgenic lines for three or more generations if not noted otherwise."

2. Are the overall transgenesis rates the same for all vectors?

As elaborated under point 1, and no construct we introduce here has any toxicity or other adverse effects on zebrafish – we selected the components based on previous validation by others (as cited, see for instance fluorophores).

3. They deploy an array of different fluorophores and argue these do not bleed into more traditional colors such as GFP. These claims are largely unsubstantiated - there are relatively few double transgenics for example. As noted in issue (1), this could be addressed by either adding data or by reducing claims as written in the text.

We appreciate the reviewer's concern.

We write in the text: "Spectral separation of mCerulean from GFP is easily achievable on common confocal and light sheet setups, either by using a 445 nm or 458 nm laser or using features of typical confocal software platforms to minimize spectral overlap (such as in Zeiss' ZEN software). We noted that depending on genetic background, mCerulean imaging on a scanning confocal microscope resulted in auto-fluorescence of skin or pigment cells, particularly in the head region of zebrafish embryos and larvae (48 hpf onwards) when visualized with an Argon laser (488 nm) (Fig 2A-F). Use of the ideal excitation laser wavelengths greatly diminished this auto-fluorescence and we have not found any significant problems with mCerulean reporter imaging at these stages. Of note, mCerulean is easily visible on a standard dissecting scope with epifluorescence equipment, ideally using blue fluorescence filters and excitation spectra. mCerulean is also excited and detectable by standard setups used for GFP fluorescence, requiring care to avoid misinterpreting mCerulean versus GFP signal in double-transgenic embryos."

For use with dissecting fluorescence scopes, such as the one we describe in our Methods, please see also our response to Reviewer 2's point 2.

4. The pineal gland transgenic reporter is interesting, but it actually looks difficult to photograph. How is it in use on stereo dissecting fluorescent screening scopes?

Please see point 3 – several of the images in the manuscript have been taken by a standard dissecting scope with fluorescence setup. The pineal reporter was selected particularly for ease of screening on standard equipment and without much training. We have now labeled instances of dissecting scope imaging as such in the figure legends (see for instance Figure 5, where we depict how the reporter looks like in a routine clutch observed with routine microscopy settings).

5. Providing clear data on the limitation of IRES use would be good for the field. Are there good tangible examples the authors can provide as they developed the prior transgenesis kit that heavily

© 2023. Published by The Company of Biologists under the terms of the Creative Commons Attribution License (https://creativecommons.org/licenses/by/4.0/).

depended on IRES expression? Some individual reports are included. But a comprehensive collection on this topic would be an excellent use of space within this manuscript.

We appreciate the reviewer's concern, but there are no published examples we can provide, simply because it didn't work well. In the past decade, we and others have performed numerous reporter tests with *IRES* constructs that failed throughout. The IRES constructs in Kwan et al. 2007 had been included by popular request at the time.

For the manuscript under review here, we present a collection of validated 2A constructs, many of which we have been sharing for several years already across the community. Our manuscript here is the first formal documentation of their application, aiming to also homogenize their description and use. We have written more extensively about the well-validated 2A peptides in our response to Reviewer 1's point 3.

In brief, 2A constructs have been used for well over a decade in several fields including in vitro reprogramming to achieve stochiometric expression levels of stringed-together transcription factors. As the 2A peptides are host factor-independent, they currently provide the best molecular tool to connect different ORFs together before their separation.

Minor:
Note: adding 2A peptide sequence can reduce the expression of the upstream ORF.

We are using the P2A from porcine teschovirus-1 2A as published by Kim et al., 2011 (https://www.ncbi.nlm.nih.gov/pmc/articles/PMC3084703/.

P2A exhibits a cleavage efficiency of nearly 1:1 as shown in Kim et al.'s Fig. 2 A and B. Further, Kim et al. show in Fig. 5A that "WB analysis exhibited that P2A has the highest cleavage efficiency in zebrafish as well, followed by T2A, E2A and F2A". Even if the two proteins are not separated due to failed cleavage, the upstream ORF would still remain read. We are therefore not clear why the reviewer anticipates that the upstream ORF would be reduced in its expression (translation).

Textual
I found the number of missing citations substantial to make reading the paper difficult to fully assess. Some examples are below. Might be simpler to find a few reviews and focus more on the science in the paper. I point out a few critical ones, but none are more important than those from Fisher 2006.

We do cite the two publications from Fisher et al., in 2006 in the first and last paragraphs of the introduction, in the results for Fig. 6, and in the Discussion. These are the appropriate parts of the manuscript where the citations belong. This is landmark work and we heavily rely on these pioneering applications in our own work.

Intro, first paragraph
The selection of examples showcasing transgenic constructs seems rather arbitrarily selected and omits a variety of classics (such as the first GATA GFP from Shuo Lin).

We have now added additional citations, yet do appreciate that our citation efforts are by no means comprehensive.

Paragraph two - Tol2 was first harnessed by Dr Koga's lab, isolated from medaka. That citation is missing here but found later in the paper (Koga 1996).

We cite Koga et al., 1996 in the last paragraph of the introduction, but have re-added Koga et al., 1996 to the second paragraph in the Introduction to appropriately cite Tol2's original isolation.

Last paragraph - the selection of citations seems off as well. Amsterdam 1995 is a great paper, but it's not generating a lot of transgenics with expression. And if the focus is on first transgenics, many other citations are missing. Might be simpler to cite far fewer rather than have a broader aperture and clearly miss critical papers.

© 2023. Published by The Company of Biologists under the terms of the Creative Commons Attribution License (https://creativecommons.org/licenses/by/4.0/).

We are fully aware that we are not able to comprehensively cite all work, and would appreciate concrete examples of what citations the reviewer thinks we have omitted (which we did not purposefully do so).
We would like to point out in this instance that we had stated “Building upon the work of many before us including, but not restricted to…”. This citation string is meant to acknowledge the actual origins of the key parts that we are using in our manuscript here, and not as whole-sale acknowledgement of transgenesis in the model per se. The references include first use of mouse beta-globin, transgenesis markers, backbones, etc. incorporated in our vector collection. We have now rephrased this to “Building upon components and applications generated by many before us...”

Given the reviewer feels a more comprehensive recap of transgenesis in zebrafish is missing, although not the aim or scope of our manuscript, we hope that our efforts motivate a brief summary article with Development. A comprehensive, historic acknowledgement of the individual steps leading to our current abilities to generate transgenic zebrafish would be immensely beneficial to the field, especially for newcomers.

Results
First paragraph: also many citations missing, including two Fisher et al 2006 key papers. And papers prior to Fisher conducting targeted enhancer trapping in zebrafish etc.

We cite both Fisher et al. papers from 2006 for Fig. 6 and other parts, as outlined above. The citations listed in this paragraph are for the first reported use (to the best of our knowledge) of the listed individual minimal promoters. Fisher et al., 2006 used *ef1a*, which to the best of our knowledge had been first used in zebrafish as minimal promoter by Meng et al., 1997 (cited as such in our manuscript).

Second decision letter

MS ID#: DEVELOP/2022/201531

MS TITLE: Next-generation plasmids for transgenesis in zebrafish and beyond

AUTHORS: Cassie L. Kemmler, Hannah R. Moran, Brooke F. Murray, Aaron Scoresby, John R. Klem, Rachel L. Eckert, Elizabeth Lepovsky, Sylvain Bertho, Susan Nieuwenhuize, Sibylle Burger, Gianluca DAgati, Charles Betz, Ann-Christin Puller, Anastasia Felker, Karolina Ditrychova, Seraina Botschi, Markus Affolter, Nicolas Rohner, C. Ben Lovely, Kristen M. Kwan, Alexa Burger, and Christian Mosimann
ARTICLE TYPE: Techniques and Resources Article

I am happy to tell you that the reviewers are happy with your revisions and your manuscript has been accepted for publication in Development, pending our standard ethics checks. The referee reports on this version are appended below.

Reviewer 1

Advance summary and potential significance to field

Kemmler et al. report the production of a new collection of Gateway compatible vectors for transgenesis in zebrafish and some other species, such as the Mexican cavefish. This work represents an expansion of the original Tol2Kit (Kwan et al., Developmental Dynamics, 2007) to expand existing functionality, including additional options for fluorophore and transgenesis marker selection, and to introduce new functionality including vectors for enhancer testing and for p2a-based protein co-expression.

© 2023. Published by The Company of Biologists under the terms of the Creative Commons Attribution License (https://creativecommons.org/licenses/by/4.0/).

Comments for the author

The authors have addressed my criticisms of the original version of the manuscript. In my opinion it is now suitable for publication as a Resource paper in Development.

Reviewer 2

Advance summary and potential significance to field

As mentioned in the firast review, I believe the resources presented here for transgenesis are going to be of great utility/imporance to the community.

Comments for the author

The authors have addressed all of my concerns; I therefore recommend the manuscript be accepted as a Techniques and Resources paper.

Reviewer 3

Advance summary and potential significance to field

This paper reports a series of new transgenesis vectors suitable for use in fish.
Their deployment in the miniTol2 vector makes these accessible to most current labs. The selected images are well-presented. The text language strongly indicates these lines show Mendelian expression patterns, an issue they argue has prevented prior tools from more regular use in the field. These vectors will likely be well-used by the research community and represent a valuable asset.

Comments for the author

The authors have addressed my main concerns.

© 2023. Published by The Company of Biologists under the terms of the Creative Commons Attribution License (https://creativecommons.org/licenses/by/4.0/).
